# Participatory modeling from a stakeholder perspective: On the influence of collaboration and revisions on psychological ownership and perceived model quality

**DOI:** 10.1007/s10270-022-01036-7

**Published:** 2022-08-23

**Authors:** Anne Gutschmidt, Birger Lantow, Ben Hellmanzik, Ben Ramforth, Matteo Wiese, Erko Martins

**Affiliations:** 1 Chair of Business Information Systems, Institute of Informatics, University of Rostock, Albert-Einstein-Straße 22, 18059 Rostock, Germany; 2 Institute of Informatics, University of Rostock, 18051 Rostock, Germany; 3 Fachhochschule des Mittelstands - University of Applied Sciences Rostock, Kröpeliner Str. 85, 18055 Rostock, Germany

**Keywords:** Participatory enterprise modeling, Collaboration, Psychological ownership, Perceived control, Perceived model quality

## Abstract

Participatory enterprise modeling is about gathering domain experts and involving them directly in the creation of models, aided by modeling experts. It is meant to increase commitment to and quality of models. This paper presents an exploratory study focusing on the subjective view of the domain experts. We investigated the influence of direct collaboration versus individual modeling, and the influence of model revisions by modeling experts on psychological ownership and perceived model quality. We chose process modeling as a particular form of enterprise modeling. Our results give hint that domain experts working individually with a modeling expert perceive model quality as higher than those working collaboratively whereas psychological ownership did not show any difference. Revisions caused changes in the subjects’ assessments only of model quality. Moreover, we will present qualitative results from interviews we led with the participants. They reveal interesting insight on how outcome and perception of the procedure and the method in both settings can be positively influenced. The interviews also emphasize the special role of the method experts who are sometimes even considered as co-owners of the model.

## Introduction

Enterprise modeling helps companies in systematically capturing knowledge about their organization, processes, responsibilities, product structures, IT systems, etc. On the one hand, models may depict a current state; on the other hand, models may be used to visualize plans of a future state [37, 38]. Models, such as business process models, can be used for simulation and deployment. Nevertheless, in many cases their actual use lies in sense making, communication and improvements within the organization [16].

Enterprise modeling may be carried out in different ways, especially with regard to knowledge elicitation. Participatory modeling means that the stakeholders are actively involved in the modeling process by gathering them, letting them discuss and create the models jointly, supported by method experts. The latter master modeling method and notation, while the stakeholders play the role of domain experts who concentrate on contributing the relevant content [41].

Participatory modeling is well suited for companies that are consensus oriented. Modeling projects may take more time with a participatory approach, but this is compensated by higher model quality and higher commitment to the models [38, 41].

In this paper, we concentrate on the subjective perceptions of domain experts toward the model they have created, i.e., feelings of ownership toward the model and perceived model quality. We claim that the stakeholders should feel that the model is *theirs*. Psychological ownership, introduced by [33], has been shown to have a positive effect on affective commitment, the desire to maintain a relationship [22, 31]. This is particularly important when a model depicts future plans the stakeholders should later help to implement.

Model quality is important when a model is meant to be used for simulation or deployment. However, it is also important to take the stakeholders’ perception of model quality into account. In consumer research, it was found that the quality of products and services has a positive influence on satisfaction, trust and loyalty [1, 18]. Thus, we additionally claim that domain experts should perceive the quality of the model as good, so that they will be satisfied with and trust the outcome.

We will address two factors that might have an influence on psychological ownership and perceived model quality. First, a participatory setting involves the collaboration of several people, i.e., several domain experts and at least one method expert. A setting where a method expert models together with only one domain expert at a time might lead to different feelings of ownership. Secondly, method experts usually refine models and present them again in a subsequent meeting, either because there was not enough time in the preceding meeting to model everything that was said or because further sources have been consulted in the meantime. So, the original model has been changed by someone else.

To sum up, we examine possible differences in psychological ownership and perceived model quality that might be caused (1) by collaboration between domain experts in a participatory setting, and (2) by revisions made by modeling experts.

To further explore what we can do to positively influence the variables, we also led individual interviews at the end of each trial. We wanted to find out what promotes or weakens the emergence of psychological ownership and what positively or negatively influences model quality from the participants’ perspective.

In the following section, we will give more explanations on participatory modeling, psychological ownership and model quality, leading to our research questions. In Sect. 3, we will describe our research method. We have conducted an experiment where groups of five persons either worked collaboratively or individually on a process model, always supported by a method expert. After a first meeting, the method expert refined the model and presented it to the participants in a second meeting. After each meeting, psychological ownership and perceived model quality were measured. A mixed analysis of variance was used to explore the data for differences concerning the two dependent variables. Qualitative content analysis was used to explore the interview materials. The results of the study will be presented in Sect. 4 and discussed in Sect. 5.

## State of the art and theoretical background

### Participatory and collaborative enterprise modeling

In enterprise modeling, there exist two main roles. On the one hand, the stakeholders, also called domain experts, usually belong to the company that requires the models. They hold the knowledge and ideas that should be captured and visualized. On the other hand, method experts, who may come from outside of the company, have the task to support the domain experts by putting the domain experts’ knowledge and ideas into models. The method experts master modeling methods and notations so that the domain experts can concentrate on the content [38, 41].

The process of creating the models can be implemented in different ways. The method experts may elicit the necessary information via interviews, by observation and by scanning documents and then, based on this information, they draw the models [38]. When method experts create the models in a team it is called collaborative modeling. The most important goal of collaborative modeling is to create complete models in a fast and accurate way [3].

Participatory modeling goes a step further by involving several stakeholders at a time. It is particularly beneficial when consensus among the stakeholders is important [10, 37]. Sandkuhl and Seigerroth characterize participatory modeling along two dimensions: the number of modelers and stakeholder involvement. With participatory modeling, a group of stakeholders and method experts collaboratively develop the models. The authors refer to all the remaining settings as conventional modeling, including bilateral modeling with one method expert and one domain expert as described above. With this definition, they do not only stress the involvement of stakeholders but also put the collaboration between the stakeholders (supported by modeling experts) to the foreground [37]. Among the method experts, further role distributions may be applied. A facilitator who leads the discussion is always present. Optionally, there is a tool operator who handles the modeling tool and a secretary who documents the modeling process [34, 38, 41]. The advantage of a participatory setting is that there is an exchange of knowledge and ideas among the stakeholders. Furthermore, conflicting perspectives that may not have been discovered or resolved in individual consultations become explicit in participatory sessions [30, 42]. This might also be the reason why Sandkuhl and Seigerroth [37] consider collaboration as part of the definition of participatory modeling as opposed to conventional modeling. As a consequence of this, participatory modeling is claimed to lead to greater commitment to the models, including the plans and ideas that may be connected to them. Additionally, it is said to lead to greater model quality as information elicitation is more exhaustive [37, 41]. However, empirical evidence is scarce as we will point out in the next section.

### Empirical studies on participatory and collaborative modeling

Most of the studies examining the effects of stakeholder participation can be characterized as case studies, such as [9, 42]. They outline positive effects and give recommendations; however, causal relationships between a participatory procedure and commitment, ownership feelings and model quality, respectively, have not been directly examined.

Luebbe and Weske conducted a study where a domain expert created a process model aided by a method expert. They used a special tangible toolkit that should make modeling easier for beginners [19]. They found that, compared to the traditional way of interviewing the domain expert and having the method expert create the model alone, the domain experts took more time thinking about the process, made more corrections and had more insights into process modeling. An influence on commitment could not be found, but the study did not use a standardized scale. Moreover, it would have been interesting to examine the effect of gathering domain experts in the sense of group model building [34].

Gjersvik et al. suggested their own procedure for participatory process modeling called modeling conference [10]. The procedure includes alternating sessions where domain experts work in sub-teams and plenary sessions where the partial results are discussed and merged. Moreover, the authors recommend that, at first, the sub-teams should be homogeneous. In later steps, more heterogeneous teams should cause a richer exchange of different perspectives. Four cases where this procedure was implemented were accompanied by empirical examinations of acceptance of the model and ownership feelings. While acceptance increased and ownership feelings did not change comparing an intermediate model with the final model for the participants, both constructs were lower for employees of the same companies who had not taken part in the conferences. The study does, however, not compare different levels of participation in the model creation for the actual participants.

Nolte et al. developed a procedure for process modeling where the participants collaborate in a first meeting followed by a phase of two weeks with only asynchronous collaboration [30]. As the authors assume some participants to be lay modelers, they let the participants only make annotations instead of real changes to the model. In a second meeting, the annotations (and responses to them) were discussed and possibly implemented into modifications of the model. The authors stated that their suggested procedure made lay modelers feel and actually be more in control of the creation process, also reflected in the participants’ rating of the model quality. Nevertheless, the study does not provide a comparison between different levels of participation either.

Other authors give hint on how to evaluate participatory modeling projects. Assuming that different methods can be used for participatory modeling, Jones et al. [14] suggest a framework for evaluating whether the method used is appropriate for the current context and for the people involved [14]. The framework includes a comparison of the participants and the project team. The evaluation is very complex and seems to aim at re-calibrating a running process of participatory modeling. Ssebuggwawo et al. claim that modeling artifacts, i.e., modeling language, modeling procedure, modeling tools and the model itself, are the drivers of the modeling process and should therefore be central constructs in the evaluation of participatory modeling projects [39]. Thus, according to the authors, it is, among other aspects, important that the participants perceive the quality of the model as good. Besides efficiency and effectiveness, so-called perceived quality of the procedure includes satisfaction and aspects such as commitment and shared understanding while the model is judged with regard to aspects called product quality, understandability, modifiability, maintainability and satisfaction [40].

To sum up, although commitment, ownership feelings and perceived quality have appeared in former publications on participatory and collaborative modeling, empirical studies that directly examine causal relationships between participation and these criteria are scarce. We want to address this research gap with the study presented in this paper. We will consider the artifacts model and modeling procedure, i.e., we will manipulate parts of the procedure, collaborative vs. individual working, and investigate effects on the participants’ rating of the procedure and the model.

### Psychological ownership

Psychological ownership means that people feel they own something, e.g., an object, a person or an idea, although they may not legally possess the target of interest [33]. In organizational contexts, it was shown to lead to positive effects such as citizenship behavior and extra role behavior [2, 31] and affective commitment [22, 31]. The latter one means that one feels the desire to maintain the relationship toward, e.g., a company [24]. Pierce and colleagues name three so-called routes that lead to psychological ownership. One must control the target, have intimate knowledge about the target, and invest the self into the target in terms of labor, time and other personal resources [32, 33].

In this paper, we will compare participatory modeling (a team of domain experts models collaboratively, supported by a method expert) with individual modeling (where the method expert creates the model together with single stakeholders). In the participatory setting, the stakeholders follow the essential parts of the creation process. They are present when the others make their suggestions. In the second setting, everyone generates their individual model and they are later confronted with others’ input which may be supporting, adding to or contradicting with their preceding input. Perceived control over the process may play a major role in this context. Being confronted with a model to which others have contributed, but who the participant has not met or communicated with, might diminish a feeling of being in control. However, one may also feel less in control when working in a team where others are faster or louder. So, in the participatory setting, some people might not be able to give as much input as in individual meetings. Moreover, some team members might not be as motivated as others. With regard to getting to know the target thoroughly, participants of a participatory modeling session may get new insights into the subject through their teammates. However, this is also possible when being confronted with a model built from several individual models. That way, the participants might get to see different pieces of knowledge although there is no synchronous exchange. Thus, it is for us to explore whether these two different settings cause any difference in psychological ownership. As it is very difficult to hypothesize about the exact influence of collaboration on feelings of ownership, we formulate an open research question:

**RQ1: Do these different settings (participatory/collaborative versus conventional/individual) cause a different level of ownership feelings toward the model?**

Please note that, with regard to the terms participatory and conventional, we refer to the definition by Sandkuhl and Seigerroth [37] where participatory includes not only the involvement of stakeholders into the model creation but also the collaboration of the modelers. Thus, conventional includes the creation of a model in sessions with only one stakeholder involved.

Another influence that has to be considered is the one of revisions by the modeling expert. The fact that one may not have been present when further parts of the model were created might induce a feeling of having less control over the model. Either additions are made from information the participants gave to the method expert, but which could not be modeled, e.g., due to a lack of time. Or, in case of individual models, parts of the refined model might even come from other domain experts. That is why we investigate the influence of revisions on psychological ownership. So, the second research question is:

**RQ2: Is the feeling of ownership toward the model different when the first model has been created and when being confronted with a refined model in a second meeting?**

In addition, we will look for a possible interaction between the two factors revision and collaboration.

Moreover, we want to explore further the emergence of psychological ownership in this particular context. That is why we want to consider the following research question:

**RQ3: Which factors influence the emergence of psychological ownership toward the model from the participants’ point of view and are there differences depending on the setting of model creation (participatory/collaborative vs. conventional/individual)?**

Lastly, perceived control is considered as a particularly important factor influencing psychological ownership. That is why we want to investigate the following research question:

**RQ4: Which factors influence the feeling of being in control during the modeling process from the participants’ point of view and are there differences depending on the setting of model creation (participatory/collaborative vs. conventional/individual)?**

### Model quality as subjective target

The quality of models is crucial. Only when a model meets certain standards, and especially the expectations of the stakeholders, will it be of use. Many approaches that try to define model quality are based on a division into quality aspects inspired by semiotics theory [29]. Moody and colleagues [26, 27] refer to that same theory when they use the terms syntactic quality, semantic quality and pragmatic quality. While the first one represents syntactic correctness with regard to a modeling language and its rules, semantic quality means that a model reflects reality correctly and as completely as necessary. Pragmatic quality is about the stakeholders understanding the model [26]. Krogstie [16] presented an extended system of model quality, the SEQUEL framework, adding empirical quality (comprehensibility of the model), physical quality (availability of models to the relevant actors), perceived semantic quality (correctness and completeness of the model from a stakeholder’s subjective point of view), social quality (stakeholders’ agreement on model interpretation) and deontic quality (meeting the goals of modeling). Krogstie and colleagues [13, 17] also scrutinized these criteria to create guidelines for modeling processes.

We agree that researchers and modeling experts should strive for guidelines that tell us how to create models of high quality and help us assess model quality. However, while the above-mentioned approaches take mostly a modeling expert’s perspective—which is absolutely important and useful, we want to regard quality from the stakeholder’s perspective. We claim that besides feeling that “the model is mine” it is also important that, after a model has been created, domain experts can say that “this model is of good quality.” This is in accordance with [40] who underline the importance of how the stakeholders perceive and rate modeling artifacts such as the model itself. Similarly, Rittgen suggested a scale with which domain experts could rate a model [36].

We assume that what domain experts will probably value the most is that the model contains all relevant information and is correct, that they think they and others can understand the model and that they have trust in the model’s syntactic correctness. These aspects are all part of frameworks and suggested quality metrics such as suggested by [6, 17, 25, 36].

A study by Heggset and colleagues [12] gave hint that there is a connection between syntactic quality and model comprehension. After models had been refined with regard to syntactic quality, the subjects would give more correct answers to questions about the models’ content. In a study about text revisions, Caspi and Blau [6] found that after others had edited the subjects’ texts, perceived quality increased whereas perceived ownership decreased. So, possibly, domain experts might value revisions of modeling experts and perceive them as an increase in model quality. In contrast to this, Nolte and Herrmann [30] reported that domain experts perceived a decreased understanding of the model later in the process. This might be because the participants in this setting were confronted with the model without the support of a method expert. Another reason could have been that the model became more detailed.

The modeling expert might not only improve syntactic quality but also add content that was named by the domain experts but had not yet been included in the model. Yet, revisions might not necessarily be perceived as positive. When several single models must be merged, this will mean that domain experts will in some cases be confronted with new content which might even contradict their views. Thus, the research question is:

**RQ5: Is there a difference in how domain experts perceive a model’s quality before and after it has been refined by a modeling expert?**

In a modeling session, where a modeling expert works with only one domain expert, the domain expert has a chance to voice all thoughts and ideas without being interrupted or distracted by other persons. In fact, although team work as in participatory modeling has its benefits, e.g., knowledge of different sources is gathered, it has its drawbacks. Team members might be more reluctant because someone more dominant or with a higher position in the organizational hierarchy might be present [43], or they may be less motivated, e.g., because they do not see their individual contribution in the overall outcome [15]. Moreover, collaboration requires more coordination concerning communication resulting in productivity blocking [7]. So, on the one hand, domain experts might be more satisfied with the model when they could work intensely with the modeling expert in an individual session than when having to assert themselves in a group of people. On the other hand, the variety of suggestions of different people might generate the impression of a better model. Thus, the next research question is:

**RQ6: Is there a difference between conventional/individual modeling and participatory/collaborative modeling with regard to perceived model quality?**

We will also investigate a possible interaction between the two factors revision and collaboration concerning perceived model qualities as their different combinations might lead to different outcomes.

Additionally, we want to further investigate influences on model quality from the participants’ point of view. That is why we also consider the following research question:

**RQ7: Which factors influence model quality from the participants’ point of view and are there differences in their assessments depending on the setting of model creation (participatory/collaborative vs. conventional/individual)?**

## Research method

### Experimental design

**Fig. 1:**
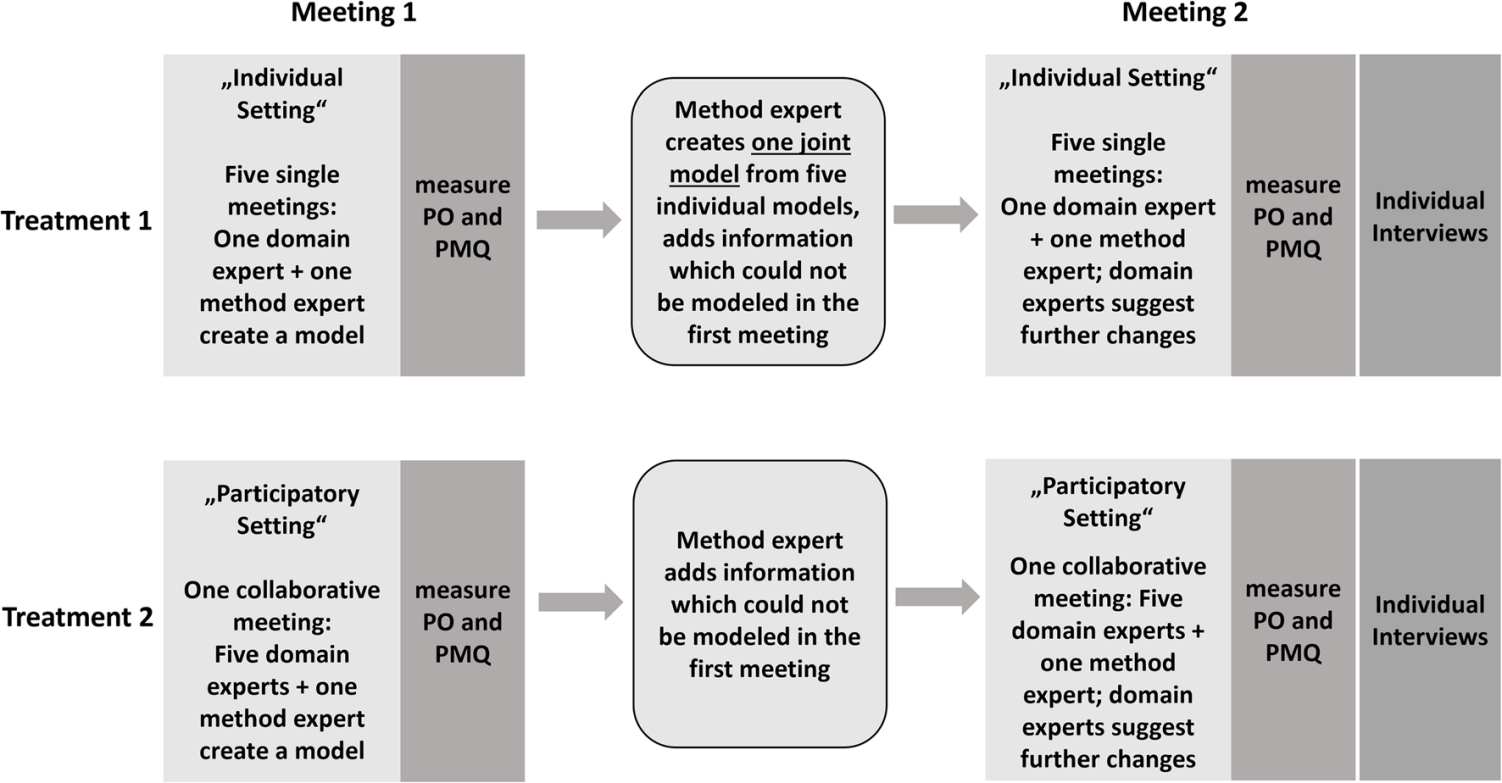
Experimental design with the treatments individual versus participatory setting, and the two meetings where each time psychological ownership (PO) and perceived model quality (PMQ) were measured

Figure 1 presents our experimental design. **The first independent variable** we considered is represented by collaboration. We either gathered a group of five persons to create a model together, aided by a modeling expert, or a modeling expert met five members of a group individually to create five separate models. The first setting represents participatory modeling whereas the second setting is to be regarded as a form of conventional modeling according to [37]. After the modeling, we asked the participants to fill out a questionnaire, assessing demographic data, modeling experience and the **dependent variables** psychological ownership and perceived model quality.

In this first meeting, we set the task to create a model that would describe the process of registering a final paper at the university, such as a bachelor thesis. We chose the Business Process Modeling Notation (BPMN) as it is a very common language for process modeling which the method experts involved in this study were familiar with. We gave a short introduction to BPMN to the participants with a brief example, covering only the most essential elements of the notation to limit complexity. After the introduction, the participants had thirty minutes time for modeling the process of interest using BPMN. Due to the Covid-19 pandemic, we did not meet the participants in person but via video conference (Zoom). The modeling was done with the software Cawemo which runs within the browser and can be used collaboratively. Cawemo is a tool for process modeling with BPMN.

For each trial, we gathered a team of five persons: two to three students (one participant in the overall sample had recently graduated, and started working), one employee familiar with the administrative part of the process and one or two employees who supervise students in writing papers. So, they were real stakeholders regarding this process. They were told that the process model could offer additional guidance to next cohorts of students who are about to start their bachelor thesis or a comparable thesis. Each group belonged to a different university.

All participants attended two modeling sessions. Thus, **the second independent variable** is represented by time of measurement using two points in time. In the second meeting, a revised model was presented to the participants. In case of the participatory setting, modeling experts would have added information that was named in the first meeting, but could not be modeled then due to a lack of time. In case of the individual modeling treatment, the modeling expert had to merge five models and presented the one resulting model again individually to the participants. The participants had 15 minutes time to suggest changes and additions. Afterward, psychological ownership and perceived model quality were again assessed using a questionnaire.

The method expert took the role of the facilitator during the meetings. They led the discussion and answered questions concerning the notation, but were not to interfere concerning the content. The participants were encouraged to draw the model themselves. In case they did not get along with the modeling tool, the method expert aided them by taking the role of a tool operator as well. Between the two meetings, it was the modeling experts’ task to create one joint model from five individual models for treatment 1, or to complete the model for treatment 2. In the latter case, the method experts used recordings of the first meeting and added information to the model that the participants had discussed. They were not allowed to add information that was not mentioned by the participants during the first meeting. They were allowed to arrange elements differently and correct spelling mistakes for better readability, and to correct possible mistakes in the modeling notation.

To sum up, this setting allowed us to investigate the influence of collaboration on psychological ownership (RQ1) and perceived model quality (RQ6), and the influence of model revisions on psychological ownership (RQ2) and perceived model quality (RQ5).

At the end of the second meeting, we led individual semi-structured interviews with each participant to further explore what influences the two considered variables. We wanted to get to know from the participants conditions and factors promoting or weakening the emergence of psychological ownership (RQ3). We were particularly interested in what influenced their perception of being in control over the modeling process (RQ4). Moreover, we wanted them to recall what had influenced model quality from their point of view (RQ7). The goal of the interviews was to get a more detailed insight in what can be done to improve the perception of the modeling procedure and the attitude toward the model.

### Measuring the dependent variables

Generally, our approach was to use several items, i.e., statements that should be rated, to measure one variable such as psychological ownership. We measured psychological ownership as dependent variable using a mix of the scales (item sets) by [44] (German version by [21]) and [6], adapted to the context of modeling. We used seven German items (statements) which had to be rated on a five-point Likert scale. Here, we present the English items: (1) This is MY model, (2) I sense that this model is OUR model, (3) I sense that this is MY model, (4) This is OUR model, (5) Most of the people that have worked on this model feel as though they own the model, (6) It is hard for me to think about this model as MINE (reversed), and (7) I feel that the model is mine, even if others contributed to its development. Thus, each statement had to be given a rating between one and five. A total score for a construct such as psychological ownership is usually obtained by calculating the average of all the ratings. However, beforehand, a factor analysis and a reliability analysis were done to check scale validity and reliability and to exclude possibly unsuitable items (statements) (see Sect. 3.3).

For the second dependent variable, we were searching for a scale which allowed accessing the subjective perception of model quality. We considered several scales, e.g., by [25, 36, 39]. We chose a very simple and short scale introduced by [6] which originally referred to self-written text, but appeared to be fitting to the modeling context nonetheless. Its main advantage is the brevity of the items enabling a quick rating. Caspi and Blau [6] reported very good reliability of their scale with a Cronbach’s alpha of 0.94 at maximum based on the data they collected in their studies. (For more details on reliability of a measuring instrument, see Sect. 3.3.) The scale comprises six items which we translated and adapted to the context of model quality. Furthermore, we used reversed formulations for two items to prevent a dominating tendency toward one side as suggested by [5]. The items ask for the model being (1) not of good quality (reverse), (2) comprehensive, (3) exhaustive, (4) well-drawn, (5) exact, and for (6) not presenting information clearly (reverse). Following the same principle as described for psychological ownership, before we could calculate an overall score (average value) from all the ratings the participants gave for each of the six attributes, we performed a factor analysis and a reliability analysis (see Sect. 3.3).

All measuring instruments were included in the questionnaire the participants had to fill out after each meeting.

### Evaluation methods

For a mixed design like ours, comprising a combination of a between-subjects design (individual vs. participatory/collaborative) and a within-subjects design (two measurements), comparisons of mean values with a mixed analysis of variance (ANOVA) is preferred. For example, the ANOVA enables us to compare the mean values of psychological ownership between the two treatments (individual vs. participatory/collaborative) to answer RQ1, and between the two meetings to answer RQ2. Beside the main effects of collaboration and point in time, the method allows the consideration of the interaction between the two factors, i.e., whether the combination of treatment and time of measurement have an effect. Its main preconditions include homogeneity of variance checked with the Levene test, and balanced treatment groups. Normal distribution of residuals is less important as long as the two former conditions are fulfilled [4].

To obtain one value for a construct such as psychological ownership (PO), a score is calculated which is the mean value of the ratings of the statements belonging to the respective measuring instrument. Beforehand, we conducted a factor analysis and reliability analysis to check the suitability of the measuring instruments for psychological ownership and perceived model quality. Factor analysis is used to check the validity of the measuring instrument, i.e., do we measure what we want to measure? Furthermore, some constructs may consist of several facets, e.g., in the past, we noticed that psychological ownership may be distinguished into collective ownership (this is ours) and individual ownership (this is mine). A factor analysis can show such multidimensionality with an output containing several factors. Items (statements to be rated) that express a similar content should be correlated with only one specific factor, thus, ideally there should be a clear assignment of related items to the same factor [28]. For estimating the reliability of the respective measuring instrument, Cronbach’s alpha was calculated. In this context, this coefficient represents the estimated accuracy of the measuring instrument; i.e., when measuring one variable with several items one should make sure the items are consistent, thus, really measuring the same variable. The accuracy could, for example, be corrupted by errors in the measurement. A typical cause for such an error is when an item, i.e., a statement that should be rated by the participants, is misunderstood [8].

When an item could not be clearly assigned to a specific factor and removing this item from the scale (item set) led to an increase of Cronbach’s alpha, we dismissed the respective item to increase validity and reliability of the measuring instrument. Subsequently, we calculated scores for the corresponding constructs of psychological ownership and perceived model quality. The scores were used as input variables for the mixed ANOVA. For all significance tests, we considered an alpha of 0.05.

To evaluate the interviews, we performed a qualitative content analysis based on [23]. First, one coder deductively determined categories according to the questions contained in the interview, i.e., the coder determined coding units in the interview transcriptions containing information about psychological ownership and, connected with it, perceived control, and factors influencing model quality from the participants’ point of view. Subsequently, the coder inductively sorted the coding units into more fine-grained categories. So, the coder came up with a system of subcategories based on the interview materials. With a second coder, we checked the validity of the subcategories and the coding units’ assignment to the subcategories. The second coder was given the categories and their definitions and categorized the coding units for PO, perceived control, and factors influencing model quality, respectively, for a second time, so we could judge the degree of congruence between first and second coder. We reached an intercoder reliability (Cohen’s Kappa) of 0.9 ($p<0.001$).

### Sample

We recruited participants from four universities with different courses of study: business information systems, computer science, specialized areas of business administration and psychology. On the whole, four groups of five persons each, 20 persons overall, took part in the study, among them 11 men and 9 women. The average age was 33.6 years ($\sigma = 11.7, \min = 20, \max = 60$). Based on a five-point Likert scale with 1 as minimum, experience with BPMN modeling was rated on average with 2.1 ($\sigma = 1.1$) for the collaboration treatment, and 2.1 ($\sigma = 1.4$) for the individual treatment. Thus, their BPMN experience was similar, but quite low on average. When the collaboration teams met in the first session, some of them knew each other already, some had met for the first time. The participants meeting the modeling expert individually knew they were part of a group and that their models would be merged to one overall model.

## Results

### Factor and reliability analyses

Although the scale measuring PO is assumed to assess a one-dimensional construct, we already found in a prior study that there was a division between individual and collective psychological ownership [11]. In this study, the participants seemed to feel this distinction, too, because it clearly shows in their rating of the PO items containing MY and OUR. The exploratory factor analysis in SPSS (principal components, Varimax rotation) showed two factors where the items “I sense that this model is OUR model” and “This is OUR model” load on one factor and the remaining items on a second factor. Nevertheless, all factor loadings were greater than 0.6 for both measurements which is a crucial criterion of the items’ suitability for measuring the respective construct [20]. The reliability analyses showed satisfying values for Cronbach’s alpha for both subscales (individual and collective quality) and all measurements (first and second meeting). For the OUR scale, Cronbach’s alpha was 0.872 and 0.912. For the MY scale, Cronbach’s alpha was 0.889 and 0.925. As a consequence, we calculated two scores, one for individual PO and one for collective PO. While we received ratings of all statements concerning PO for the first questionnaire, in the second questionnaire, the ratings of one group in treatment 1 for one statement concerning individual PO are missing. In these cases, we calculated the value of individual PO based on the remaining four instead of five ratings for these participants.

Caspi and Blau’s quality measure is originally one-dimensional [6]. Each factor analysis for the two measurement data sets showed, however, a division of items into two factors. One item (The model is not of good quality) was separated addressing general quality whereas the other items refer to specific characteristics. The item “The model does not present information clearly” loaded on factor one in the first measurement and on factor two in the second measurement, apparently being ambiguous. Cronbach’s alpha increases (1) from 0.777 to 0.853, and (2) from 0.709 to 0.759 when leaving out the item of general quality. It increases even more when removing the above-mentioned ambiguous item, to 0.885 and 0.851, respectively. We decided to use two variables: a variable consisting of only the item measuring general quality and a score calculated from average of the participants’ ratings of the items addressing the characteristics comprehensive, exhaustive, well-drawn and exact. Table 1 shows the descriptive statistics of the resulting variables, separated for the two factors collaboration and point in time of measurement.

**Table 1:** Descriptive values of psychological ownership (PO) and of perceived model quality (PMQ), separated by collaboration (yes/no) and the two measurements M1 and M2

Collaboration	Individual PO		Collective PO		General PMQ		Specific PMQ	
	$\mu$	$\sigma$	$\mu$	$\sigma$	$\mu$	$\sigma$	$\mu$	$\sigma$
*M1*								
Yes	2.2	1.2	4.5	0.8	3.7	1.1	3.3	0.9
No	3.1	1.1	3.9	1.0	3.9	1.4	4.2	0.8
Overall	2.6	1.2	4.2	1.0	3.8	1.2	3.7	0.9
*M2*								
Yes	2.1	1.0	4.1	1.1	4.3	0.7	3.7	0.6
No	2.9	1.3	4.0	0.8	4.1	1.4	4.7	0.4
Overall	2.5	1.2	4.0	0.9	4.2	1.1	4.2	0.7

### Analyses regarding psychological ownership

#### Quantitative analysis

According to the Shapiro Wilk test, not all variables showed normal distributions. The ANOVA is, however, said to be robust against the violation of this condition if the data show homogeneous variances and are balanced [4], which was the case for our data set.

For the individual PO, the mixed ANOVA resulted in nonsignificant effects for time of measurement ($F = 0.4$, $df = 1$, $p = 0.5$), for the interaction of the two factors ($F = 0.1$, $df = 1$, $p = 0.7$) and for the collaboration treatment ($F = 3.4$, $df = 1$, $p = 0.08$).

For the collective PO, the mixed ANOVA again showed only nonsignificant effects, i.e., for time of measurement ($F = 0.6$, $df = 1$, $p = 0.4$), for the interaction of the two factors ($F = 1$, $df = 1$, $p = 0.3$) and for the collaboration treatment ($F = 0.8$, $df = 1$, $p = 0.4$).

Thus, neither the different measurements nor collaboration nor the combination of the two factors showed a difference in PO.

#### Qualitative analysis

In the interviews, we asked the participants about their **feeling of ownership** toward the model. In particular, we wanted to know whether they felt that it was MY model or OUR model and why. Twelve persons, including nine from the participatory setting, said they felt it was OUR model because **everyone had contributed** to the outcome. Some even said it was teamwork, e.g., “Because we did it in group work. And I think we all got involved. And everyone has a part in it.” While these statements contained a “we” or “everybody,” four participants from the individual setting noticed their **individual part** in the overall solution, e.g., “I would see me as part of [...] my influences are in there.” Two participants from the participatory condition and four participants from the individual condition explicitly assigned a part of the ownership to the **modeling experts** who had, between the two meetings, completed and/or merged models, e.g., “Who was really modeling now, that was your team, wasn’t it? That’s why I would say, yes, that’s 80 percent your model and 20 percent only ours.” There were two persons who had taken part in the individual settings who either were barely aware of **other participants’ contributions** or they saw other’s contributions just as a confirmation of their work. Six participants, including five from the individual setting, said they could not feel it was MY model because the **model looked different**, in terms of different wording and new content parts. Two persons, one from each setting, said that **drawing the model themselves** would have had a positive influence on their feelings of ownership. One of these participants even stated that everyone had their own style of modeling with BPMN: “Through the drawing itself and, well, notations or not, so - that’s why in the area of “MY model.” Because everyone draws a little differently with BPMN processes.” One participant (individual) said the model would belong to those who would receive the final outcome. Another participant (individual) said that he/she would not say it was MY model because of their socialization explaining this with “I am from the east”, probably indicating that people who have been brought up in the eastern part of Germany (formally GDR) would have a different attitude toward this. An overview of all the categories specifying the emergence of PO with a number of mentioning of at least two can be found in Table 2.

As an introduction to the topic of **control** in the interview, we asked the participants to rate how much control and influence they had on the creation of the final model. (It turned out that some participants reacted very sensitively to the term control and found it very negative, so we started using the term influence as an addition.) We asked them in particular why they felt low or high control and evaluated the answers they had given in this context.

**Table 2:** Categories from the qualitative content analysis with overall frequencies $>1$, showing frequency of mentioning for both treatments and overall

Category	Individual	Participatory	Sum
*Psychological ownership*			
Everyone contributed	3	9	12
Method experts’ contribution	4	2	6
The model looks different	5	1	6
My individual part	4	0	4
Other domain experts’ contribution	2	0	2
Drawn myself	1	1	2
*Perceived control*			
Everyone is heard	2	7	9
Influence of method experts	4	3	7
The model looks different	5	1	6
Experience with modeling or tool	0	4	4
Time constraints	0	3	3
Awareness of others’ influence	3	0	3
Recognition of own contributions	3	0	3
Content-related contribution	1	2	3
Unclear roles	0	2	2
Other circumstances, e.g., team structure	0	2	2
Method, e.g., time alone to study the model	1	1	2
Personality	1	1	2
Did not model	1	1	2
Did model	0	2	2
*Influences on quality*			
Diversity of people involved	2	4	6
Lack of time	2	2	4
Application domain	3	0	3
Modeling experience	0	3	3
Involvement	0	2	2
Notation	2	0	2
Support by method experts	0	2	2
Agree on a common goal	0	2	2
Time to study the model on one’s own	0	2	2

The participants described circumstances that made them take action such that they exerted influence. Two persons, both from a participatory setting, mentioned that responsibilities or **roles were not clear**, e.g., after having waited for someone to start, one participant took control of the situation and started to model. Three persons from a participatory team said they felt that **time constraints** had a negative influence on their taking control, e.g., they had not enough time to create a more comprehensive model. The aspect of being able to freely speak their mind and **being heard** and considered by everyone was noticed mostly in the participatory setting (seven out of nine comments), e.g., “Everyone could say something at any time. It was then discussed and then modeled.” One participant from each setting would have preferred a different method where they had more **time alone to study the model** or that they would have created a first model on their own and then discussed it in a team. Four participants in the participatory setting said that their experience or lack of **experience concerning modeling notation or tool** was an influencing factor. In both settings, one participant said that **personality** would play a role, i.e., whether one dares voicing ideas in front of strangers or contradicting others.

Two participants from the participatory setting named **other circumstances** having an effect on taking action: the composition of the team and technical difficulties. The participants also mentioned reasons why they felt a low influence on the model. Three participants, exclusively from the individual setting, reported that their awareness that **others had also contributed** to the outcome had diminished their feeling of having influence. One participant from each setting said they did not feel to be in control as they **had not modeled themselves**. Seven participants, including four from the individual setting, perceived that the **method experts had a significant extent of control** over the creation process. One participant from the participatory setting said that some of his or her ideas were not included in the model, while five participants in the individual setting noticed that the final **model looked different** from the original model, e.g., because additions were made.

Three participants, exclusively from the individual setting, mentioned that they felt they had had some influence on the model because they **recognized aspects** in the final model they had originally contributed. The participants named concrete actions with which they took control. Two participants from the participatory setting and one participant from the individual setting mentioned their **content-related contributions**; two participants from the participatory setting mentioned that they **had modeled**. An overview of all the categories regarding perceived control that were mentioned more than once among all participants can be found in Table 2.

### Analyses regarding perceived model quality

#### Quantitative analysis

As with PO, normal distribution was not always fulfilled for the perceived model quality variables, but the Levene test showed homogeneity of variance and the data set was balanced.

For the variable addressing general perceived model quality, there was a significant main effect for time of measurement ($F = 7.2$, $df = 1$, $p = 0.015$), but not for collaboration ($F = 0$, $df = 1$, $p = 1$) and not for the interaction of the factors ($F = 1.8$, $df = 1$, $p = 0.2$).

For the second variable, summarizing specific perceived quality attributes, there was a significant main effect for time of measurement ($F = 6.8$, $df = 1$, $p = 0.018$), and for collaboration ($F = 12.2$, $df = 1$, $p = 0.003$), but not for the interaction of the factors ($F = 0.2$, $df = 1$, $p = 0.7$).

To sum up, there was a difference between the two measurements of both perceived general quality and the variable measuring specific perceived aspects of model quality. The latter one also showed a difference caused by collaboration versus working individually. Having a look at the descriptive statistics in Table 1, we can see that perceived model quality is higher after the second meeting than after the first meeting, and specific model quality is perceived as higher by those participants who did not work collaboratively.

#### Qualitative analysis

In the interview, we had asked the participants what had influenced the quality of the final model from their opinion. Three participants, only from individual settings, said that the quality depends on the domain context, i.e., how complex the issue to be modeled is. Two persons (participatory) said that personal **involvement** in the topic was beneficial, e.g., “[...] probably also the degree of personal involvement with regard to this topic. So, is it something I’m totally far away from and somehow have to imagine it?”

Some participants mentioned aspects that had an influence on what the model looked like. Two participants from the individual setting said that the **notation** had an influence on model quality. One participant would have preferred colors, the other praised the possibility of modeling roles in BPMN. One participant from the individual setting liked that the model was not drawn by hand but by using a **computer-supported tool**, i.e., a web application.

Four persons in the participatory setting and two persons in the individual setting said the **diversity of people involved** had a positive influence on model quality, e.g., “Because several people from different areas can bring in thoughts from different perspectives. And have taken into account various experiences [...].” Two persons from the participatory setting mentioned the **support by modeling experts** as a positive influence, e.g., “And then I think what also helped was that when there were small questions, you always answered directly.” Three persons (participatory setting) mentioned **modeling experience** as an influencing factor, i.e., the participants did not feel prepared or skilled enough.

**Lack of time** was named as a negative influence by four persons, two from each setting, e.g., one participant said that it would not have been possible to create a high-quality model within the given time frame.

Some participants underlined the importance of the **modeling method** and suggested some improvements. Everyone should **agree on a common goal** (two participants in the participatory setting) and **clarify the distribution of roles**, i.e., who can make which content-related contribution and who will draw the model (one participant in the participatory setting). Furthermore, one participant suggested a certain procedure where first, the content should be discussed and afterward, the model should be created. Two participants from a group that had worked in a participatory setting said they would have preferred to have **some time to study the model on their own**. An overview of all the categories describing influences on perceived model quality that were mentioned more than once among all participants can be found in Table 2.

## Discussion

### Summary and interpretation

We have conducted a study on enterprise modeling that focused on the subjective view of the domain experts. We were interested in how they perceive quality of and ownership feelings toward a model they helped creating depending on different settings. We compared a participatory setting where domain experts modeled collaboratively supported by a method expert with a setting where method experts worked with domain experts in individual meetings. Furthermore, we examined how the domain experts’ assessments changed after the model had been revised by method experts. In this section, we want to discuss our findings on psychological ownership and perceived model quality and present our major conclusions.

*Psychological ownership* With regard to psychological ownership, we have not found any differences caused by collaboration between the domain experts **(RQ1)** nor by revisions made by modeling experts **(RQ2)** in our statistical analysis. Consequently, no matter whether the participants worked individually or collaboratively, their ownership feelings toward the model did not differ significantly nor did revisions by modeling experts cause any changes in that feeling. Considering the descriptive statistics, it becomes clear that the participants make a distinction between individual and collective psychological ownership. Apparently, the feeling that the model is common property is more predominant.

One possible explanation of why we did not find any differences in psychological ownership, especially after revisions, could be that the subject to be modeled was not controversial enough. Caspi and Blau had shown that editing of one’s text by other people decreased psychological ownership [6]. In our study, there is just a slight and nonsignificant decrease in three of the four groups with regard to psychological ownership. This effect might have been stronger if the issue to be modeled had involved different opinions and more profound differences in the knowledge of the participants. We seem to have chosen a process which was very clear to the participants. There were only few small parts that some participants had not been aware of before.

On the whole, collective psychological ownership shows a high average value in this study comparing it to the greatest possible value of 5. Even the participants working individually showed higher collective ownership feelings than individual ownership feelings, which is especially interesting with regard to the first meeting where the output model was based on only the one respective participant’s input. Unfortunately, we did not assess to whom the participants were referring when they said the model is “OUR” model. We suspect that the participants considered the method experts as co-owners which is supported by our qualitative findings from the interviews (see below).

One could assume that drawing the model oneself would have an influence on the emergence of psychological ownership. The fact that many teams in the participatory setting modeled themselves did not lead to higher psychological ownership. This is in accordance with a former study showing that if modeling experts take on the task of drawing, this will not undermine the emergence of psychological ownership [11].

Through interviews, we investigated possible influences on the emergence of psychological ownership **(RQ3)** and the feeling of being in control of the modeling process **(RQ4)** from the participants’ point of view. With regard to both questions, we also wanted to know whether these assessments differed depending on whether the participants had worked individually or collaboratively.

Most frequently, the participants said it was important that everyone could contribute to the process to develop a feeling of ownership. Many participants also said that they felt in control because everyone was heard, e.g., could voice their ideas or opinions. This was mentioned far more often by those who had worked collaboratively (see Table 2). Maybe, these participants have been more aware of this because they were facing a situation where they were one of several members in a group in contrast to talking alone to the method expert in an exclusive meeting.

Moreover, the participants found that it was detrimental to feeling ownership over the model and being in control when the model looked different after its revision by the method expert, especially in the individual treatment. In the individual setting, the participants do not see what the other participants had contributed in their first meeting. The latter may not even be perceived as co-owners.

Connected to this, we encountered statements from six persons, including four from the individual setting, that assigned part of the ownership to the method experts who had completed and/or refined the model between the two meetings. The two persons from the participatory setting who considered the method experts as co-owners belonged to the same team. Compared to the other participatory team, they had more trouble with the time constraints and discussed more than they modeled. Consequently, a lot of the modeling happened between the meetings, executed by the method experts. Only in this group we encountered statements that the lack of time had a negative influence on feeling control. It is likely that the more is modeled in the absence of the participants, the greater they will see the part the method experts have in the ownership of the model.

With regard to perceived control, mostly participants from the individual treatment saw it as problematic when the model looked different after the revision. It seems that it is important to witness as much of the modeling as possible to develop a feeling of control. Furthermore, in the same participatory team that was mentioned above, participants had a critical view with regard to the method experts. Method experts were attributed with a considerable extent of control over the model creation. It is to be examined whether we should weaken this perception and how this could be done.

Only in the participatory treatment, participants said that modeling and tool experience had an influence on perceived control. As mentioned above, it was mostly the participants from the participatory setting who were modeling themselves. Yet, usually only some of the team members were active. Other participants with less experience might have been more aware of their alleged deficits. Either one has to make sure in the participatory setting that all participants can model, e.g., by training them or, which is more realistic, by letting them take on very easy actions and encouraging them to try things out. The alternative would be to use an in individual setting where the single participants cannot be “outrun” by more experienced participants.

Statements from both individual and participatory settings give hint that we might favor approaches such as [30, 35] where participants either work in alternating phases of group and individual work or they collaborate asynchronously with a modeling tool. It would make actions by the method experts as well as own contributions and contributions by other domain experts more traceable. Using special collaborative tools would also give the participants more time alone to think about the model and the issue to be modeled, as desired by some of the participants on our study.

Our findings from the interviews are in accordance with the theory on collective psychological ownership [32], i.e., the experience of working together, as it was the case in the participatory setting, seems to have a positive influence on collective psychological ownership. With regard to developing a feeling of being in control, we learned from the interviews that, especially in participatory settings, it is important to give enough time, to clarify responsibilities and roles, and to facilitate sessions in a way that everybody is heard and considered.

*Perceived model quality* With respect to perceived model quality, we found that we had to split the measuring instrument we used in two dimensions. The original measuring instrument was suggested to be one-dimensional. A factor analysis on our data led, however, to two factors. Looking at the factor loadings, we found that the statement capturing general quality (the model is not of good quality (reversed)) correlated with one factor while all the other statements that related to specific model characteristics (the model is comprehensive, exhaustive, well-drawn and exact) correlated with another factor. This leaves open the question of what the participants’ understanding of general quality includes and if there are further characteristics of a model that are important to domain experts. We will have to reconsider existing approaches on measuring model quality, e.g., [17, 25, 40], as a basis. Nevertheless, we have decided to continue our evaluations with a variable called general quality and another one we called specific quality. The latter was calculated based on the average value of the ratings referring to the specific model characteristics.

Both quality variables showed a significant difference with regard to revisions by the modeling experts. Perceived quality tended to be higher in the second meeting, i.e., after the method expert’s revision **(RQ5)**. What the participants might have noticed is that the models were extended, syntactically revised and reformatted. It seems that this is perceived as an improvement. This is in accordance with previous findings by [6]. From this, we conclude that the participants value the support they get from the modeling expert as very high.

With regard to the quality variable comprising specific characteristics, the intense exchange with the modeling expert in the individual setting seems to make a difference; i.e., the participants rated the quality higher than those participants in the participatory setting **(RQ6)**. In the first setting, the participants could describe the process and the modeling experts would help them translate everything into model elements step by step. So, there is probably an additional value of bilateral modeling in more thorough explanation of what is modeled and how.

The interviews helped us shed some light on possible influences on perceived quality and differences between an individual and a participatory setting with regard to that **(RQ7)**. Most importantly, the participants said that the diversity of persons involved had a positive influence on model quality. There were more people in the collaborative setting (4) than in the individual setting (2) who stated this advantage. In both settings, the lack of time was seen as a negative influence on quality. Three participants from the participatory setting said the quality was also depending on modeling experience of the participants. This makes clear how important it is to choose a setting that minimizes barriers for less experienced participants. In individual settings, this might be easier to implement because the participants will not be able to compare their skills with others. In the participatory setting, one must design facilitation and interaction between the participants in a way that will foster equality. Moreover, simple modeling notations and tools should be used.

The interviews have shown us how important the modeling procedure is especially in participatory settings, e.g., goals and role distribution have to be clarified to take the burden of coordinating the teamwork. Some participants said that model quality would have been better if they had had time to work and think alone which again speaks for the approach of Nolte of alternating collaborative and individual working [30].

*Major conclusions* Based on our results, we draw three major conclusions. First, to support the emergence of psychological ownership, it is not necessary to organize modeling sessions with single stakeholders. Participatory settings seem to be equally suited to trigger feelings of collective ownership. If a model should depict a future state or goals the stakeholders have to agree on, single bilateral meetings would probably be even counterproductive. Second, modeling experts are of great importance to the domain experts in both settings. As stated earlier by other authors [38, 41], for participatory modeling, it is advisable to have a facilitator and a tool operator support the domain experts. The facilitator has the important task to lead the discussion and make sure everyone is heard. But the domain experts also trust in the method experts’ modeling expertise which will probably increase the domain experts’ confidence and trust in the outcome. Third, as more and more work is done by the method experts, even without interfering in content-related aspects and, in particular, when this happens in the absence of the domain experts, the role of the method experts as co-owners becomes larger and larger. This is not always desirable, especially when method experts are hired consultants from outside the company.

### Limitations and remedies

The sample we considered in this study was small and can only give first insights. However, it has to be noted, that the implementation of this study was connected with a great effort in time both for the participants and for the researchers. The sample comprised persons who had never created a model before as well as some persons with modeling experience. A greater sample with more inexperienced participants would increase generalizability.

Our outcomes, especially concerning psychological ownership, have certainly been influenced by the task we set. With a task where more discussion and new ideas would be required, the participants, especially in the individual setting, might have assessed the situation and the model differently. Participants would probably have been confronted with more new model parts that might even be in conflict to their knowledge or opinions.

The participants’ motivation might have been limited as the output of the study did not have any consequences for them. This could have had an influence on how they assessed the model. Nevertheless, our participants had to work on a modeling task set in their real everyday life and we expected them to be willing to share their experience.

A typical requirement of an experiment is to keep all factors constant except for the independent variables to increase internal validity, i.e., for tracking the influence of the independent variables on the dependent variables. That is why we kept the modeling task and context constant. This, however, restricts external validity, i.e., generalizability of the results. That is why it is important to replicate the study in the future in other application fields with different modeling tasks.

From the experiment, we experienced that in participatory modeling, more time must be given because discussion takes more time. This is a known characteristic of participatory modeling [37]. Nevertheless, our intention was to keep the conditions in the treatment groups as similar as possible. That is why we set the same time limit for all groups. Future studies should reconsider how to make the conditions more comparable.

Furthermore, we would have liked to implement a more standardized procedure of who was modeling during the meeting. This is, however, not a trivial task. If we had determined that only the method expert should model, this could have undermined the motivation of those participants who were eager to model themselves. Moreover, the method expert as the sole tool operator could have become a bottleneck in the modeling process [36]. On the other hand, some participants were anxious or just not experienced enough to handle a modeling tool on their own. For future studies, we would suggest a procedure where simple modeling actions, e.g., adding descriptions to prepared activity components, should be taken by the participants, while more complex ones, e.g., adding events in the process model, should be taken by a method expert.

The fact that we had different method experts involved in the study might have had an influence on the model and consequently on how the participants rated the model. We, however, found that the processes suggested by the participants did not show profound differences. So, the merging of the models was not as complex as it might be with subjects of discussion where very different opinions are involved. In future studies, either one constant method expert should work on the model or a strict set of rules on how to revise and merge models should make sure that this part of the study is standardized.

Finally, due to the Covid-19 pandemic, we could not meet the participants in person. We cannot rule out that this could have had a general influence on the communication between all actors.

### Implications for practice and future research

Despite a small sample and some limitations in the experimental setting, the study presented in this paper offers an informative insight into how domain experts perceive a model under certain conditions. We were especially interested in the impact of a participatory setting on the assessment of model quality and feelings of ownership and control. Both variants we examined, participatory/collaborative and individual working, have their advantages and disadvantages.

Individual settings may sometimes seem more practical. No gatherings of several domain experts, including, e.g., negotiating joint appointments or coordinating and facilitating teamwork, have to be organized. Moreover, the participants may benefit from having the opportunity to individually discuss the model with a method expert. The intense exchange with the method expert may eventually lead to a greater perceived model quality. This individual working might, however, also lead to more complications. How should the modeling expert proceed when some domain experts give conflicting information? In future studies, we want to address more controversial issues to be modeled.

To sit down and work on a model with every domain expert in single meetings and then merge different models will also cost a method expert a lot of time. One might use an incremental proceeding where every domain expert adds further information to the same model. But who decides on the right sequence of domain experts to meet when, e.g., a given model might influence or restrict participants in their thinking?

Our interviews give hint that when the process of model creation is not completely traceable for the participants they will increasingly consider the method experts as co-owners to whom they hand over control over the modeling process. **Thus, in individual settings, every participant’s contributions to the model must be traceable, e.g., by visualization.**

Participatory modeling sessions are time-consuming for the participants and they require a lot of effort from the method experts with regard to facilitation. Our results confirm how important it is that **the facilitator of a participatory session makes sure that everyone is heard** and can voice their opinion. Another aspect that must be taken into consideration in participatory settings, in contrast to individual settings, is that special care must be taken to **prevent the impression that others can contribute better to the model generation because of their experience.** Possible barriers to join the modeling must be minimized, e.g., by using a simple notation and tool. Yet, on the whole, sessions that focus on teamwork and in which everyone is heard and can equally contribute seem to promote collective ownership feelings.

As mentioned above, participatory modeling sessions require more time. Our strategy to let the participants discuss and later let method experts add this content to the model seemed to have a negative influence on psychological ownership and perceived control. **Participants should generally witness as much of the model creation process as possible or content parts must be traceable to their originators.**

**Participatory sessions, besides promoting collective ownership, offer the possibility of exchanging different perspectives.** According to our participants, this positively influences model quality. Nevertheless, there were participants in the participatory setting who would have preferred some time alone to examine and think about the model. This is in accordance with suggestions by Nolte et al. [30] which we should further investigate. An interesting research question would be whether dealing with the model on one’s own, possibly between modeling sessions, is a form of familiarizing with the model that could again promote psychological ownership. Nolte et al. mostly considered control as a driving force [30].

The study has generally underlined the **importance of method experts with regard to model quality.** Their contributions seem to lead to more trust in the model’s quality. Our results give hint that the method experts should take on the task of modeling and make sure that the participants can follow the model creation process. From our participants’ perspective, their support in terms of modeling expertise and facilitation activities influences model quality.

Future work should involve the exploration of **method experts as co-owners.** We would like to investigate consequences of this perception and how this feeling might be mitigated.

Finally, it must be noted that this study considered just the point of view of the domain experts. Based on the data we have already collected, we also plan to compare the objective quality of a model with the perceived model quality.

## References

[1] Anderson EW, Sullivan MW (1993). The antecedents and consequences of customer satisfaction for firms. Mark. Sci..

[2] Avey JB, Avolio BJ, Crossley CD, Luthans F (2009). Psychological ownership: theoretical extensions, measurement and relation to work outcomes. J. Organ. Behav. Int. J. Ind. Occup. Organ. Psychol. Behav..

[3] Barjis, J.: CPI modeling: collaborative, participative, interactive modeling. In: Proceedings of the 2011 Winter Simulation Conference (WSC), pp. 3094–3103 (2011).10.1109/WSC.2011.6148009

[4] Bühner M, Ziegler M (2009). Statistik für Psychologen und Sozialwissenschaftler. Pearson Studium—Psychologie.

[5] Bühner M (2006). Einführung in die Test- und Fragebogenkonstruktion.

[6] Caspi A, Blau I (2011). Collaboration and psychological ownership: how does the tension between the two influence perceived learning?. Soc. Psychol. Educ..

[7] Diehl M, Stroebe W (1987). Productivity loss in brainstorming groups: toward the solution of a riddle. J. Pers. Soc. Psychol..

[8] Döring N, Bortz J (2016). Forschungsmethoden und Evaluation in den Sozial- und Humanwissenschaften.

[9] Eitzel, M., Solera, J., Hove, E.M., Wilson, K., Ndlovu, A.M., Ndlovu, D., Changarara, A., Ndlovu, A., Neves, K., Chirindira, A., et al.: Assessing the potential of participatory modeling for decolonial restoration of an agro-pastoral system in rural Zimbabwe. Citizen Sci. Theory Pract. **6**(1) (2021). 10.5334/cstp.339

[10] Gjersvik, R., Krogstie, J., Folstad, A.: Participatory development of enterprise process models. In: Information Modeling Methods and Methodologies, pp. 195–215 (2005). 10.4018/978-1-59140-375-3.ch010

[11] Gutschmidt A, Sauer V, Schönwälder M, Szilagyi T, Pańkowska M, Sandkuhl K (2019). Researching participatory modeling sessions: An experimental study on the influence of evaluation potential and the opportunity to draw oneself. Perspectives in Business Informatics Research.

[12] Heggset, M., Krogstie, J., Wesenberg, H.: The influence of syntactic quality of enterprise process models on model comprehension. In: CAiSE Forum, pp. 89–96 (2015)

[13] Heggset M, Krogstie J, Wesenberg H, Gaaloul K, Schmidt R, Nurcan S, Guerreiro S, Ma Q (2015). Understanding model quality concerns when using process models in an industrial company. Enterprise, Business-Process and Information Systems Modeling.

[14] Jones NA, Perez P, Measham TG, Kelly GJ, d’Aquino P, Daniell KA, Dray A, Ferrand N (2009). Evaluating participatory modeling: developing a framework for cross-case analysis. Environ. Manag..

[15] Karau SJ, Williams KD (1993). Social loafing: a meta-analytic review and theoretical integration. J. Pers. Soc. Psychol..

[16] Krogstie J (2012). Model-Based Development and Evolution of Information Systems: A Quality Approach.

[17] Krogstie J (2016). Quality of Business Process Models.

[18] Lie D, Sudirman A, Butarbutar M (2019). Analysis of mediation effect of consumer satisfaction on the effect of service quality, price and consumer trust on consumer loyalty. Int. J. Sci. Technol. Res..

[19] Luebbe A, Weske M, Mouratidis H, Rolland C (2011). Tangible media in process modeling—a controlled experiment. Advanced Information Systems Engineering.

[20] MacCallum RC, Widaman KF, Zhang S, Hong S (1999). Sample size in factor analysis. Psychol. Methods.

[21] Martins E (2010). Psychological Ownership in Organisationen: Explorative Untersuchung der Antezedenzen und des Entstehungsprozesses.

[22] Mayhew MG, Ashkanasy NM, Bramble T, Gardner J (2007). A study of the antecedents and consequences of psychological ownership in organizational settings. J. Soc. Psychol..

[23] Mayring, P.: Qualitative Inhaltsanalyse, Grundlagen und Techniken, 11., aktualisierte und überarb. aufl. edn. Beltz. Philipp Mayring (2010)

[24] Meyer JP, Herscovitch L (2001). Commitment in the workplace: toward a general model. Hum. Resour. Manag. Rev..

[25] Moody DL, Ling TW, Ram S, Li Lee M (1998). Metrics for evaluating the quality of entity relationship models. Conceptual Modeling—ER ’98.

[26] Moody, D.L.: Theoretical and practical issues in evaluating the quality of conceptual models: current state and future directions. Data Knowl. Eng. **55**(3), 243–276 (2005). 10.1016/j.datak.2004.12.005**(Quality in conceptual modeling)**

[27] Moody, D.L., Sindre, G., Brasethvik, T., Solvberg, A.: Evaluating the quality of information models: empirical testing of a conceptual model quality framework. In: 25th International Conference on Software Engineering, 2003. Proceedings, pp. 295–305 (2003). 10.1109/ICSE.2003.1201209

[28] Moosbrugger H, Kelava A (2020). Testtheorie und Fragebogenkonstruktion. Springer-Lehrbuch.

[29] Morris, C.W.: Foundations of the theory of signs. In: International Encyclopedia of Unified Science, pp. 1–59. Chicago University Press (1938)

[30] Nolte, A., Herrmann, T.: Facilitating participation of stakeholders during process analysis and design. In: De Angeli, A., Bannon, L., Marti, P., Bordin, S. (eds.) COOP 2016: Proceedings of the 12th International Conference on the Design of Cooperative Systems, 23–27 May 2016, Trento, Italy, pp. 225–241. Springer, Cham (2016)

[31] O’Driscoll MP, Pierce JL, Coghlan AM (2006). The psychology of ownership: work environment structure, organizational commitment, and citizenship behaviors. Group Organ. Manag..

[32] Pierce JL, Jussila I (2011). Psychological Ownership and the Organizational Context: Theory, Research Evidence, and Application.

[33] Pierce JL, Kostova T, Dirks KT (2003). The state of psychological ownership: integrating and extending a century of research. Rev. Gen. Psychol..

[34] Renger M, Kolfschoten GL, de Vreede GJ, Dietz JLG, Albani A, Barjis J (2008). Challenges in collaborative modeling: a literature review. Advances in Enterprise Engineering I.

[35] Rittgen, P.: Coma: A tool for collaborative modeling. In: CAiSE Forum, vol. 344, pp. 61–64 (2008)

[36] Rittgen, P.: Collaborative modeling of business processes: A comparative case study. In: Proceedings of the 2009 ACM Symposium on Applied Computing, SAC ’09, pp. 225–230. Association for Computing Machinery, New York (2009). 10.1145/1529282.1529333

[37] Sandkuhl, K., Seigerroth, U.: Participative or conventional enterprise modelling? Multiple-case analysis on decision criteria. In: Rowe, F., Amrani, R.E., Limayem, M., Newell, S., Pouloudi, N., van Heck, E., Quammah, A.E. (eds.) 28th European Conference on Information Systems—Liberty, Equality, and Fraternity in a Digitizing World, ECIS 2020, Marrakech, Morocco, June 15–17, 2020 (2020)

[38] Sandkuhl K, Stirna J, Persson A, Wißotzki M (2014). Enterprise Modeling: Tackling Business Challenges with the 4EM Method. The Enterprise Engineering Series.

[39] Ssebuggwawo, D., Hoppenbrouwers, S., Proper, H.: Collaborative modeling: towards a meta-model for analysis and evaluation (2010)

[40] Ssebuggwawo D, Hoppenbrouwers S, Proper HA (2013). Applying AHP for collaborative modeling evaluation: experiences from a modeling experiment. Int. J. Inf. Syst. Model. Des. (IJISMD).

[41] Stirna J, Persson A (2018). Enterprise Modeling—Facilitating the Process and the People.

[42] Stirna J, Persson A, Sandkuhl K, Krogstie J, Opdahl A, Sindre G (2007). Participative enterprise modeling: experiences and recommendations. Advanced Information Systems Engineering, No. 4495 in Lecture Notes in Computer Science.

[43] Torrance EP (1954). The behavior of small groups under the stress conditions of “survival”. Am. Sociol. Rev..

[44] Van Dyne L, Pierce JL (2004). Psychological ownership and feelings of possession: three field studies predicting employee attitudes and organizational citizenship behavior. J. Organ. Behav. Int. J. Ind. Occup. Organ. Psychol. Behav..

